# Ultrasound parameters of early pregnancy and Doppler indices of blood vessels in the placenta and umbilical cord throughout the pregnancy period in sheep

**DOI:** 10.1186/s12917-022-03424-z

**Published:** 2022-08-30

**Authors:** Angelika Brzozowska, Tomasz Stankiewicz, Barbara Błaszczyk, Pavitra Chundekkad, Jan Udała, Natalia Wojtasiak

**Affiliations:** 1 West Pomeranian University of Technology in Szczecin, Faculty of Biotechnology and Animal Husbandry, Department of Animal Reproduction Biotechnology and Environmental Hygiene, 29 Klemensa Janickiego Street, 71-270 Szczecin, Poland; 2 Department of Biology, University of Victoria, 3800 Finnerty Road Victoria BC, Victoria, V8P 5C2 Canada

**Keywords:** Placenta, Umbilical cord, Arteries, Veins, Corpus luteum, Doppler ultrasound, Pregnancy, Sheep

## Abstract

**Background:**

Ultrasonography is one of the most important techniques that enable the detection and monitoring of pregnancy. One such study using this technique is the assessment of the hemodynamics of fetal and umbilical blood vessels.

However, there is little data on blood flow in the placentomes, which is the basic structural unit of the sheep’s placenta. Therefore, the aim of this study was to determine the Doppler parameters in the arterial vessels of the caruncles, cotyledons and the umbilical cord as well as measuring venous flow rates during the entire gestation period of the sheep. Additionally, the usefulness of various other ultrasound parameters in the early diagnosis of pregnancy in sheep was analyzed.

**Results:**

Most of the Doppler parameters in umbilical, cotyledonary and caruncular arteries were significantly correlated with the day of pregnancy (*p* < 0.01). In the early stages of pregnancy, the peak systolic velocity (PSV), regardless of the location of the artery, was significantly lower than that in the later stages of pregnancy (*p* < 0.01). PSV was also found to be significantly higher in the umbilical artery than in the cotyledonary and caruncular arteries (*p* < 0.01).

Until the 50th day of pregnancy, the end diastolic velocity (EDV) was not found in the umbilical and cotyledonary arteries. EDV was significantly higher in the caruncular arteries than in the cotyledonary and umbilical arteries (*p* < 0.01). The resistance index (RI) and pulsatility index (PI) in the early stages of pregnancy were found to be significantly higher than that in the later stages of pregnancy (*p* < 0.01). The RI and PI were significantly lower in the caruncular arteries than in the arteries of the cotyledons and umbilical cord (*p* < 0.01). In the umbilical vein, all Doppler parameters were observed to be significantly higher than those in the placentomal veins (*p* < 0.01 or *p* < 0.05). Using transrectal ultrasound, pregnancy was detected between 20 and 28 days after mating. The ovaries were observed to have corpora lutea, the diameter of which was fairly consistent from the 17th to the 56th day of pregnancy.

**Conclusions:**

It has been demonstrated that both the location of the arterial vessel in the placental-umbilical circulation and the gestational age have a significant impact on hemodynamic parameters. The results also provide new insights about the blood flow in caruncular and cotyledonary arteries, which could contribute to a more holistic understanding of hemodynamic changes in the placentas of sheep. Analyzing haemodynamic parameters in the umbilical and placental veins are preliminary studies in sheep, but it could inspire further research in this field. Furthermore, the research conducted confirms the practicality and convenience of transrectal ultrasonography in the early diagnosis of pregnancy in sheep and also indicates that the identification and imaging of the corpus luteum using B-mode ultrasonography can be a very early and simple method of confirming effective mating in sheep.

## Background

Real-time ultrasound is one of the most important techniques for detecting and monitoring pregnancy in sheep. These studies take into account the characteristic images of the uterus, the presence of the embryo and the embryonic vesicle, the embryo’s heartbeat, and the presence of placentomes [1–4]. During the detection of pregnancy using ultrasound, attention is also paid to the presence of the corpora lutea on the ovaries. In pregnancy, the corpus luteum formed after ovulation does not undergo luteolysis [5]. The detection of early pregnancy is of great importance in the management of a herd, especially since about 30–50% of embryos do not survive this initial period [6]. Early detection of pregnancy also makes it possible to begin the study of intrauterine development as soon as possible, especially since the pregnant sheep is a valuable biomedical model [7–11]. One of the earliest processes that determine the fate of the pregnancy is the development of functional uteroplacental and fetoplacental circulation. In sheep, placental angiogenesis begins as early as day 18 [12, 13]. The development of the placenta continues throughout pregnancy and is closely related to the development of blood vessels in the placenta, since increased blood flow is necessary for meeting the needs of the growing fetus and for the proper exchange of materials between the mother and the fetus [3, 14]. In sheep, the placenta is of the cotyledonous type, consisting of several dozen placentomes [15]. Each placentome is a functional unit composed of the maternal part (caruncle), formed from the endometrium covering the uterine papillae, and the fetal part (cotyledon), formed by the union of the avascular chorion and the vascularized allantois [8, 16]. Studies on the architecture of the placenta in sheep have shown that maternal vascularization is formed by branches of uterine radial arteries that penetrate the caruncle from its base and then extend along the convex surface of the caruncle. These arteries penetrate the placenta creating numerous branches and an extensive network of capillaries. The vein pattern is similar, but in the opposite direction to that of the arteries [17]. On the other hand, cotyledonary vessels enter and leave the cotyledons from the region of the placentome hilum [16, 17]. They are usually single arterial vessels, which at the area of the hilum, divide into lateral vessels and then branch out to form a capillary network [17]. Conversely, the number of veins leaving the cotyledons is usually greater due to the presence of anastomoses between blood vessels from adjacent placentomes. This vascular system is clearly visible in the postpartum fetal membranes [15]. Oxygenated and nutrient-rich blood flows from the placenta to the fetus through the umbilical veins, and deoxygenated blood returns to the placenta through the arteries [16]. In sheep, the umbilical vascular system is made up of two arteries and two veins. Along the umbilical cord, these vessels do not fork, but in the chorioallantoic area, they separate into two umbilical trunks, each of which consists of one artery and one vein. This phenomenon is well documented in postpartum morphometric research of sheep membranes [15]. There is increasing evidence that disturbances in the placental-fetal circulation causes abnormalities in fetal development, negatively affect fetal growth and lead to low birth weight [18, 19] and this increases the risk of morbidity and mortality in the early postnatal stage [20, 21]. Therefore, it is crucial to monitor the placental-fetal blood circulation throughout pregnancy. Currently, an increasingly frequently used technique in these studies is Doppler ultrasonography, which is an effective, non-invasive tool that provides information on the characteristics of vascularization and blood flow. Studies conducted so far in sheep have focused mainly on blood flow in the umbilical, uterine and fetal arteries [3, 4, 22]. Doppler indices obtained from these studies have provided clinically useful information. However, there is little data available that helps characterize blood flow in the blood vessels that are responsible for forming direct vascularization of the placentomes. Research evaluating blood flow indices in venous vessels also appears to be interesting, especially since the analysis of the Doppler spectrum in the umbilical vein may be a useful parameter to consider in the assessment of the proper development of the fetus [23]. Therefore, the aim of this study was to determine and compare the Doppler parameters in the arterial vessels of the caruncles, cotyledons and umbilical cord during the entire gestation period in sheep. The flow rates in the placentomal and umbilical veins were also determined. Additionally, the relevance of various ultrasound parameters in the early diagnosis of pregnancy in sheep was also analyzed.

## Results

Pregnancy was detected between the 20th and 28th day post mating (22.21 ± 2.35) based on the uterine appearance and the presence of the embryo and embryonic sac, in the examined sheep.

**Fig. 1:**
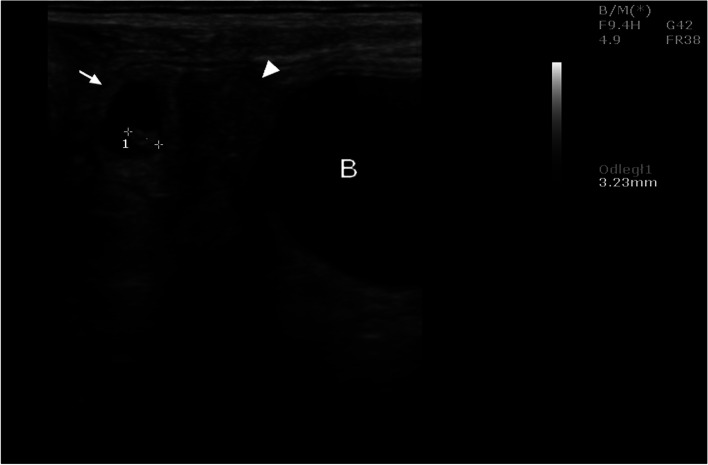
Ultrasound image of the cross section of a sheep uterus in B-Mode on the 21st day of pregnancy. Arrow - cross-section of the uterus, arrowhead - corpus luteum, 1 - embryo, B - urinary bladder

**Fig. 2:**
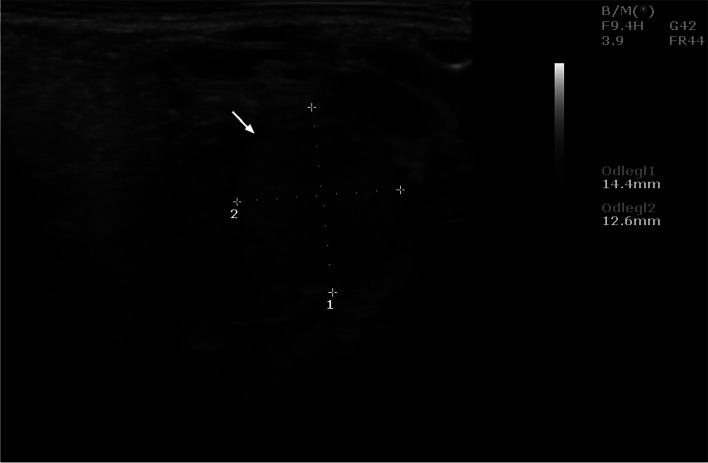
Ultrasound image of the corpus luteum of a sheep in B-Mode on day 19 of pregnancy. Arrow – corpus luteum

**Fig. 3:**
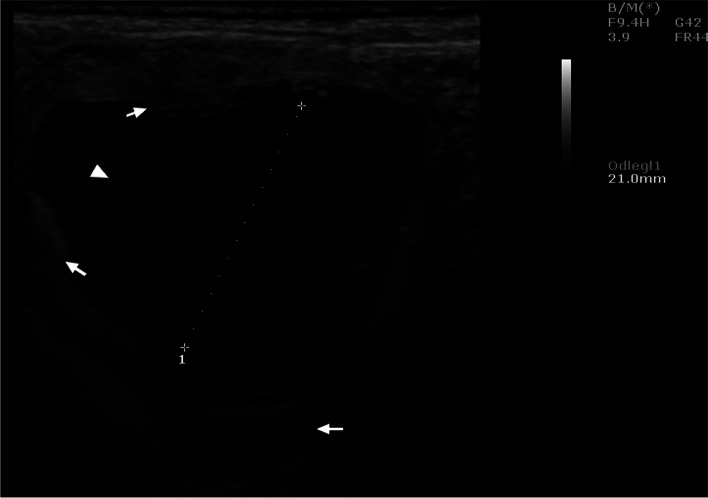
Ultrasound image of the placenta of a sheep in B-Mode on day 21 of pregnancy. Arrows - placenta, arrowhead - wall of the embryonic sac.

**Fig. 4:**
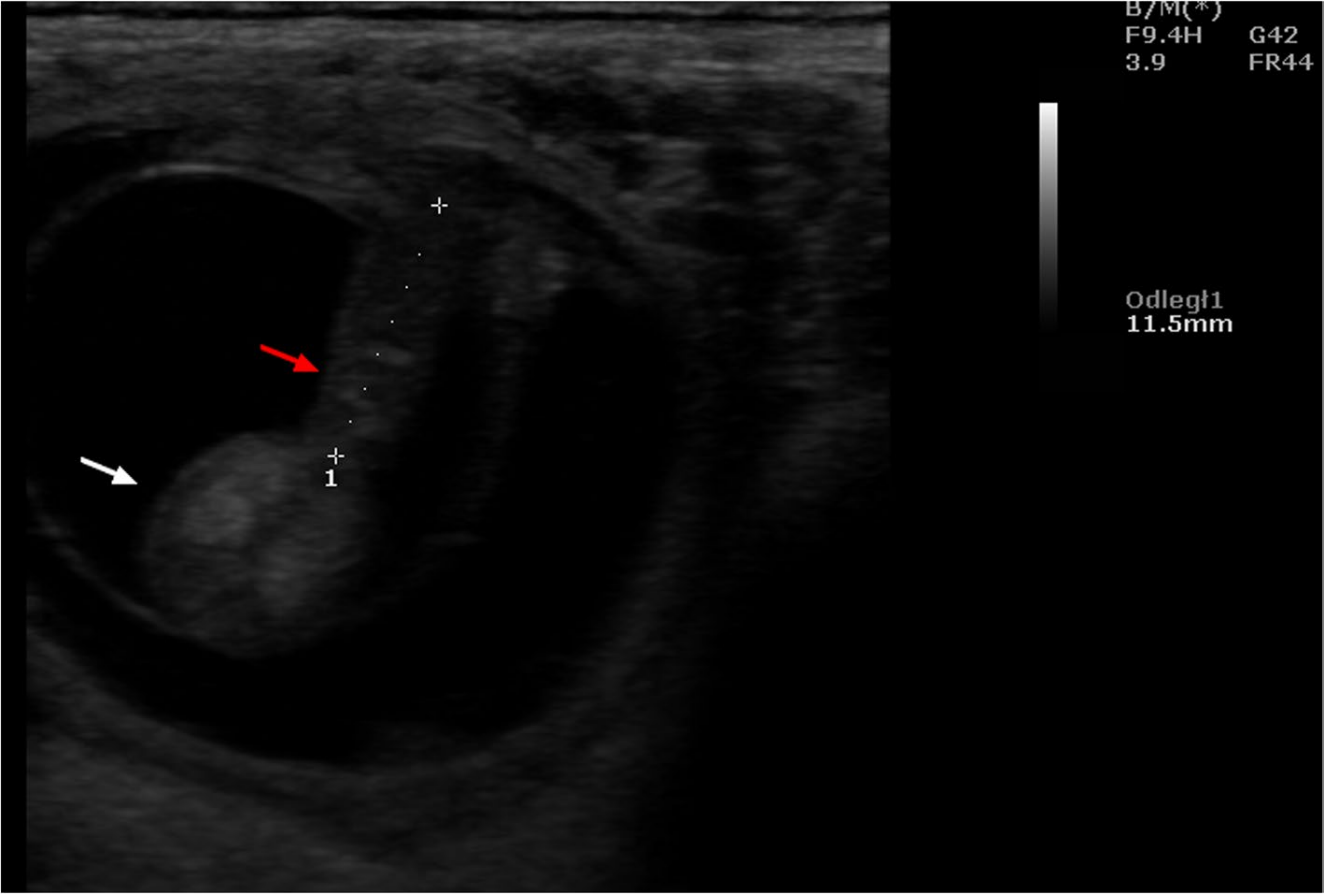
Ultrasound image of the umbilical cord of a sheep in B-Mode on day 35 of pregnancy. Red arrow - umbilical cord, white arrow - embryo, arrowhead - embryonic vesicle sac.

Ultrasound examinations carried out during the aforementioned interval showed that the uterus was oval in shape with clearly visible hypoechoic structures in its cross-section. On day 21, the embryos were visible (Fig. 1), and on day 25 - the gestational sac. Succeeding this observation, ultrasound evaluation of the ovaries performed from the 17th day onwards, indicated the presence of corpora lutea, which persisted throughout the following weeks of pregnancy. The corpora lutea were visible as gray echogenic oval structures without a round anechoic central cavity. A selected image of the corpus luteum is shown in Fig. 2. By day 56, the diameters of the corpora lutea were similar to that of the previous days, but in the days following, it was observed to have decreased significantly. The mean values of the examined biometric parameters are presented in Table 1. The placentomes and umbilical cord were first visible at 30 ± 2.15 and 33 ± 1.53 days of gestation, respectively. The ultrasound images of the placentomes and umbilical cord in this period are shown in Figs. 3 and 4. The umbilical cord diameter and length during this period were 5.28 ± 0.54 and 10.76 ± 0.62 mm, respectively, while the diameter of the first visualized placentomes was 7.73 ± 0.69 mm.

**Table 1:** Mean (± SD) of the corpus luteum diameter, uterine cross-section, embryo and amniotic sac length at the beginning of pregnancy in sheep (*n* = 16)

Days of pregnancy	Parameters			
	Diameter of corpus luteum (mm)	Cross section of uterus (mm)	Length of embryo (mm)	Length of gestational sac (mm)
17–24	13.98 ± 1.83^A^	11.29 ± 2.82^A^	2.67 ± 0.67^A^	–
25–32	13.21 ± 1.61^A^	25.35 ± 3.00^B^	7.16 ± 8.61^B^	20.83 ± 0.47^A^
33–40	12.99 ± 0.56^A^	40.21 ± 7.94^C^	26.50 ± 6.05^C^	30.50 ± 6.42^B^
41–48	13.02 ± 1.26^A^	50.53 ± 3.58^D^	37.35 ± 4.16^D^	39.90 ± 4.61^C^
49–56	13.67 ± 1.01^A^	–	–	–
57–64	8.87 ± 1.00^B^	–	–	–

A - indicates that the mean values in rows marked with different alphabets (A/ B/ C/ D) differ at *p* < 0.01

Tables 2, 3, 4, 5 and 6 show the mean values of Doppler parameters of blood flow in the placental and umbilical arteries in pregnant sheep. Table 2 shows the mean PSV values. In the umbilical artery, in all the examined periods of pregnancy, PSV values were significantly higher than that in the placental arteries. Table 3 shows the mean EDV values. During the period from 35 to 50 days of pregnancy, no EDV was found in the measurements made in the umbilical artery and in the cotyledonary arteries. A positive value of this parameter was noticed only in subsequent stages of pregnancy. In the caruncular arteries, EDV was significantly higher than that in the cotyledons and in the umbilical cord, in all stages of pregnancy. Also PSV/EDV differed significantly depending on the location of the arterial vessel (Table 4). In the umbilical artery and cotyledons, this ratio could only be determined after day 55 of pregnancy. Table 5 shows the mean RI values. In caruncular arteries, RI was significantly lower than that in the cotyledons and umbilical cord, in all examined periods of pregnancy.

**Table 2:** Mean (± SEM) values of PSV (cm/s) from placental and umbilical arteries in pregnant sheep (*n* = 16)

Location of the artery	Period of pregnancy				
	(35–50 days)	(55–70 days)	(75–90 days)	(95–110 days)	(130–145 days)
Umbilical cord	22.69 ± 1.34^A^	22.26 ± 0.74^A^	26.15 ± 0.53^Aa^	27.79 ± 1.23^a^	31.24 ± 1.43^a^
Cotyledon	10.33 ± 0.17^B^	13.18 ± 0.61^B^	14.49 ± 0.76^Ba^	22.25 ± 0.79^b^	22.87 ± 3.32^b^
Caruncle	11.07 ± 1.34^B^	14.34 ± 1.19^B^	17.86 ± 0.51^b^	26.2 ± 2,32^a^	29.18 ± 3.25^b^

A, a - indicate that mean values in the rows marked with different alphabets differ at *p* < 0.01 and *p* < 0.05, respectively

**Table 3:** Mean (± SEM) EDV values (cm/s) from placental and umbilical arteries in pregnant sheep (*n* = 16)

Location of the artery	Period of pregnancy				
	(35–50 days)	(55–70 days)	(75–90 days)	(95–110 days)	(130–145 days)
Umbilical cord	0.00	1.93 ± 0.23^A^	4.25 ± 0.72^Aa^	6.91 ± 0.66^a^	9.07 ± 0.92^a^
Cotyledon	0.00	0.04 ± 0.02^B^	3.30 ± 0.34^Ab^	6.10 ± 0.41^a^	12.10 ± 1.13^b^
Caruncle	2.10 ± 0.71	7.91 ± 0.92^C^	9.13 ± 1.74^B^	10.32 ± 1.85^b^	12.21 ± 1.21^b^

A, a - indicate that mean values in the rows marked with different alphabets differ at *p* < 0.01 and *p* < 0.05, respectively

**Table 4:** Mean (± SEM) values of PSV/EDV from placental and umbilical arteries in pregnant sheep (*n* = 16)

Location of the artery	Period of pregnancy				
	(35–50 days)	(55–70 days)	(75–90 days)	(95–110 days)	(130–145 days)
Umbilical cord	NO	8.67 ± 0.85^A^	9.56 ± 1.57^A^	4.60 ± 0.42^a^	4.22 ± 0.58^a^
Cotyledon	NO	12.03 ± 2.81^B^	12.62 ± 4.42^B^	3.78 ± 0.33^b^	1.77 ± 0.48^b^
Caruncle	0.95 ± 0.32	3.99 ± 1.10^C^	4.84 ± 0.76^C^	3.23 ± 0.23^b^	2.42 ± 0.51^b^

A, a - indicate that mean values in the rows marked with different alphabets differ at *p* < 0.01 and *p* < 0.05, respectively

NO – incalculable value

**Table 5:** Mean (± SEM) RI values from placental and umbilical arteries in pregnant sheep (*n* = 16)

Location of the artery	Period of pregnancy				
	(35–50 days)	(55–70 days)	(75–90 days)	(95–110 days)	(130–145 days)
Umbilical cord	1.00	0.95 ± 0.01^A^	0.78 ± 0.08^A^	0.76 ± 0.03^a^	0.70 ± 0.04^A^
Cotyledon	1.00	1.00	0.75 ± 0.03^A^	0.67 ± 0.03^b^	0.35 ± 0.12^B^
Caruncle	0.87 ± 0.04	0.36 ± 0.08^B^	0.42 ± 0.09^B^	0.56 ± 0.12^b^	0.52 ± 0.13

A, a - indicate that mean values in the rows marked with different alphabets differ at *p* < 0.01 and *p* < 0.05, respectively

**Table 6:** Mean (± SEM) PI values from placental and umbilical arteries in pregnant sheep (*n* = 16)

Location of the artery	Period of pregnancy				
	(35–50 days)	(55–70 days)	(75–90 days)	(95–110 days)	(130–145 days)
Umbilical cord	3.01 ± 0.12^A^	2.79 ± 0.12^A^	1.65 ± 0.09^A^	1.48 ± 0.13^a^	1.17 ± 0.11^a^
Cotyledon	2.26 ± 0.02^Ba^	2.55 ± 0.14^A^	1.37 ± 0.10^A^	1.40 ± 0.06^a^	0.64 ± 0.17^b^
Caruncle	1.66 ± 0.17^Bb^	0.73 ± 0.15^B^	0.79 ± 0.11^B^	0.91 ± 0.11^b^	0.89 ± 0.09^a^

A, a - indicate that mean values in the rows marked with different alphabets differ at *p* < 0.01 and *p* < 0.05, respectively

**Fig. 5:**
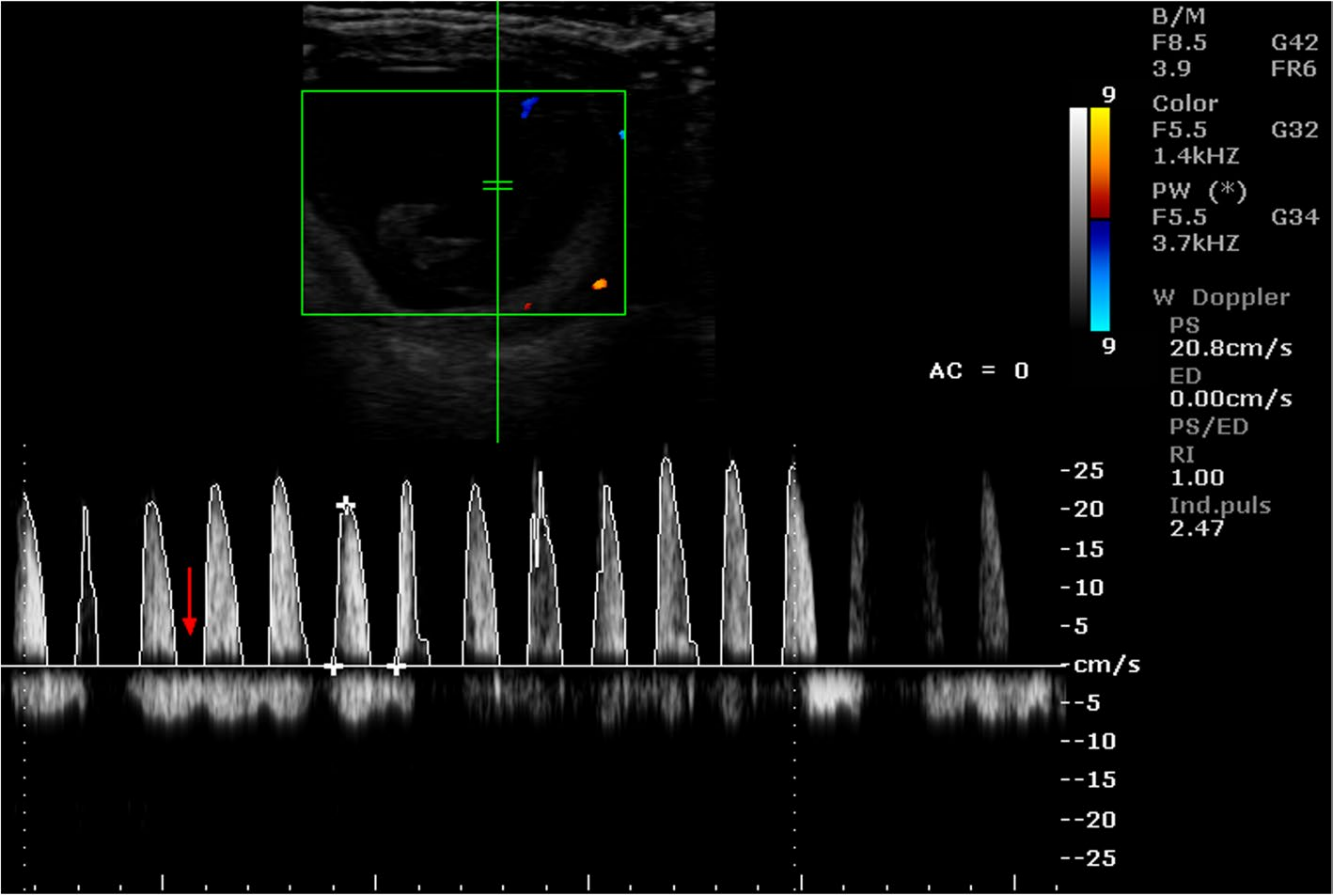
Color Doppler ultrasound image showing hemodynamic measurements from the umbilical artery of a sheep on day 35 of pregnancy (PS - peak systolic velocity; ED - end-diastolic velocity; RI - resistance index; Ind. puls - pulsatility index). Red arrow – no ED

**Fig. 6:**
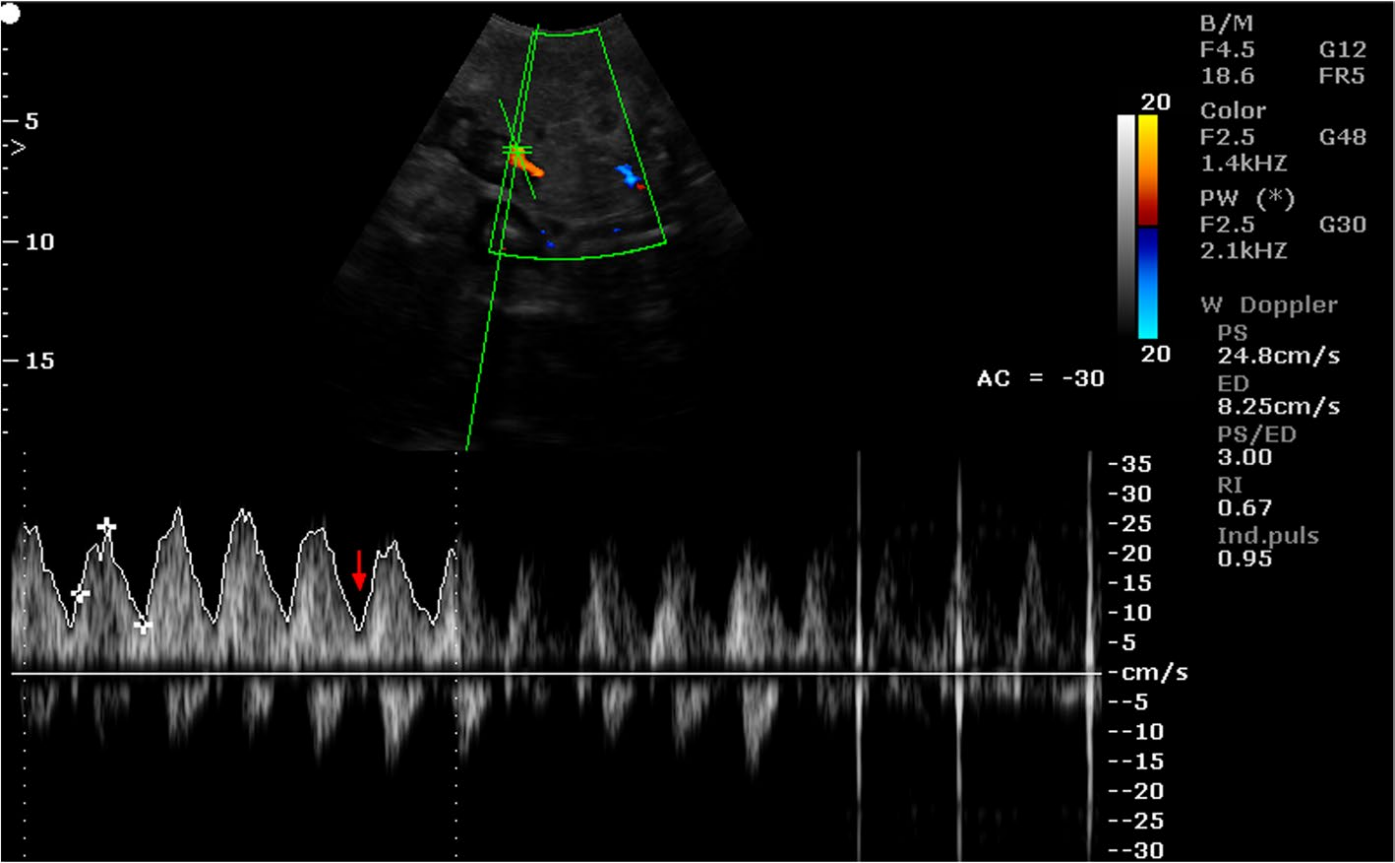
Color Doppler ultrasound image showing hemodynamic measurements from the umbilical artery of a sheep on day 130 of pregnancy (PS - peak systolic velocity; ED - end-diastolic velocity; RI - resistance index; Ind. puls - pulsatility index). Red arrow – the presence of ED

**Fig. 7:**
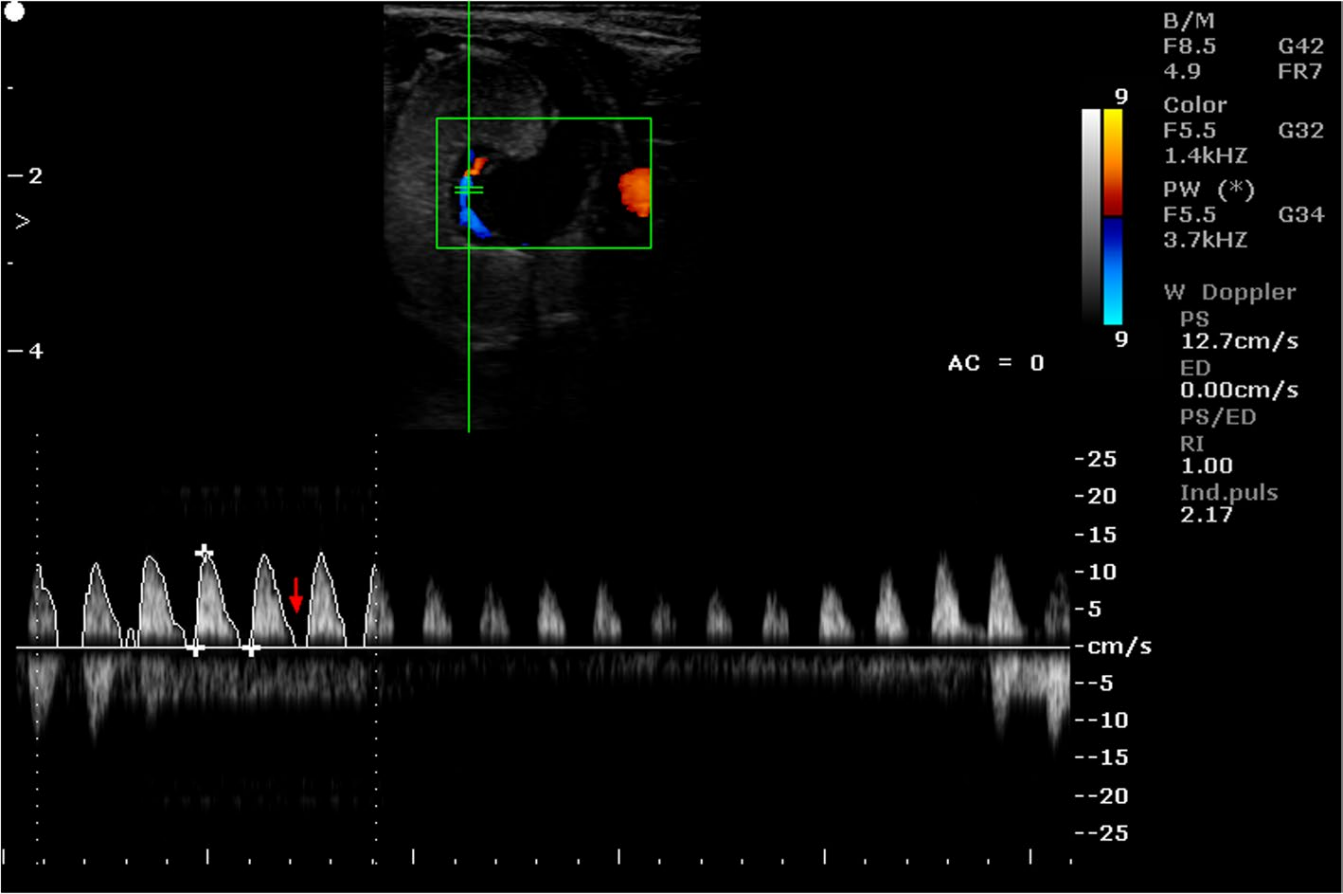
Color Doppler ultrasound image showing hemodynamic measurements from the cotyledonary artery of a sheep on day 60 of pregnancy (PS - peak systolic velocity; ED - end-diastolic velocity; RI - resistance index; Ind. puls - pulsatility index). Red arrow – no ED

**Fig. 8:**
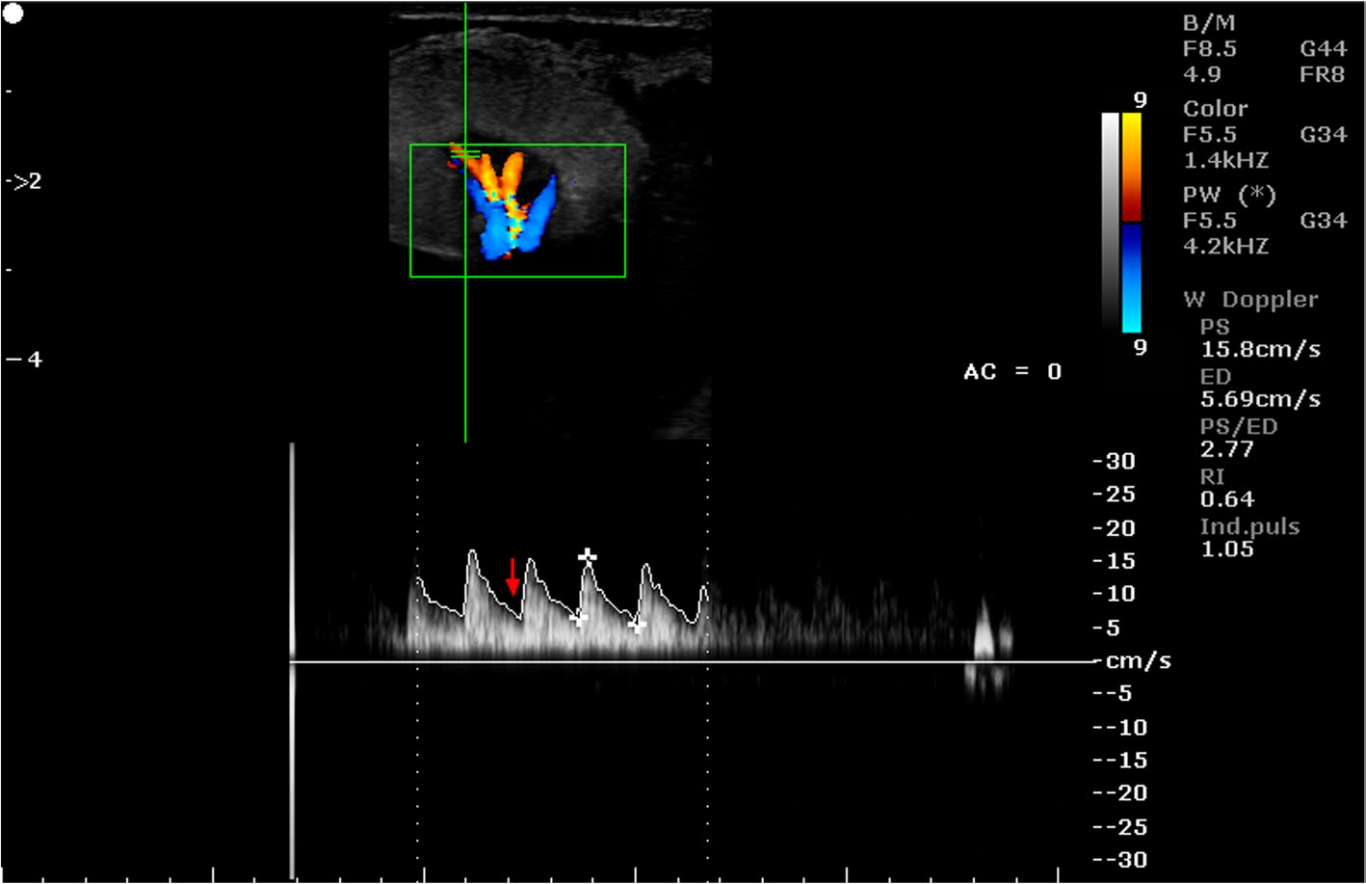
Color Doppler ultrasound image showing hemodynamic measurements from the cotyledonary artery of a sheep on day 95 of pregnancy (PS - peak systolic velocity; ED - end-diastolic velocity; RI - resistance index; Ind. puls - pulsatility index). Red arrow – the presence of ED

**Fig. 9:**
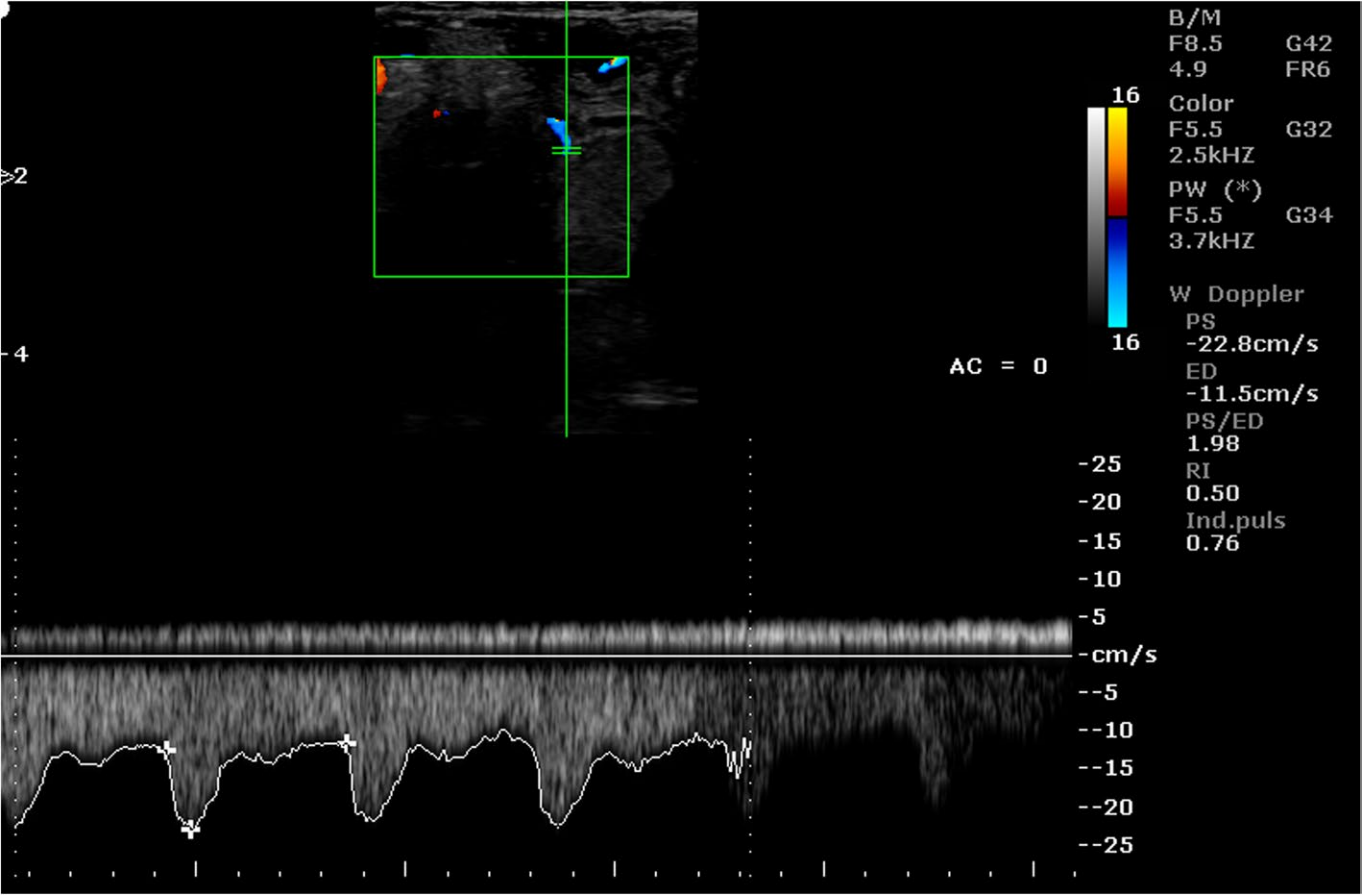
Color Doppler ultrasound image showing hemodynamic measurements from the caruncular artery of a sheep on day 95 of pregnancy (PS - peak systolic velocity; ED - end-diastolic velocity; RI - resistance index; Ind. puls - pulsatility index)

**Fig. 10:**
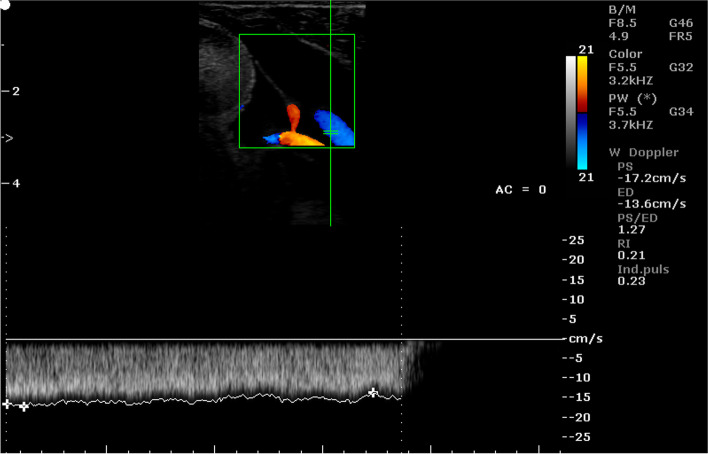
Color Doppler ultrasound image showing hemodynamic measurements from the umbilical vein of a sheep on day 90 of pregnancy (PS - peak systolic velocity; ED - end-diastolic velocity; RI - resistance index; Ind. puls - pulsatility index)

**Fig. 11:**
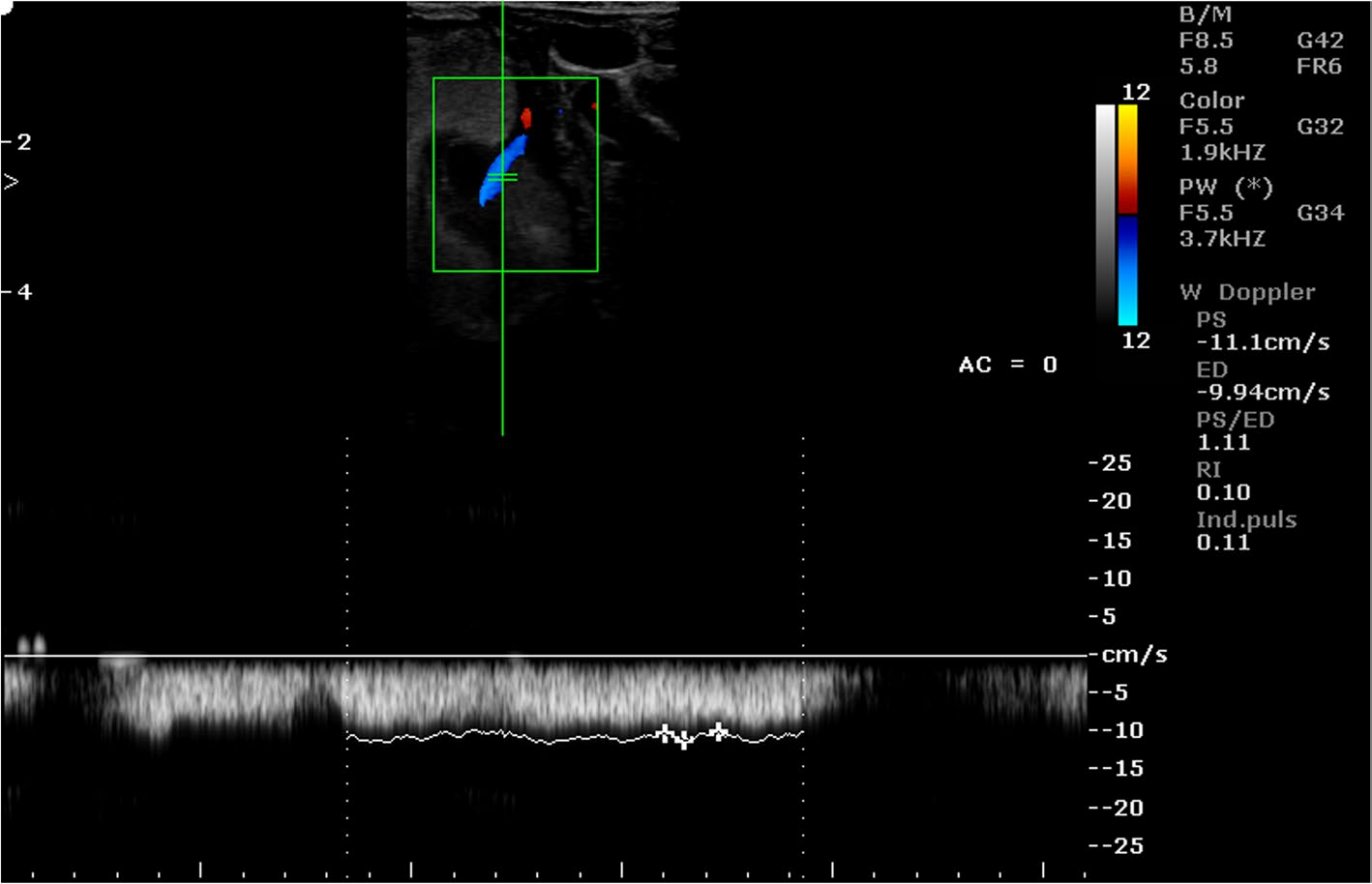
Color Doppler ultrasound image showing hemodynamic measurements from the umbilical vein of a sheep on day 86 of pregnancy (PS - peak systolic velocity; ED - end-diastolic velocity; RI - resistance index; Ind. puls - pulsatility index)

**Fig. 12:**
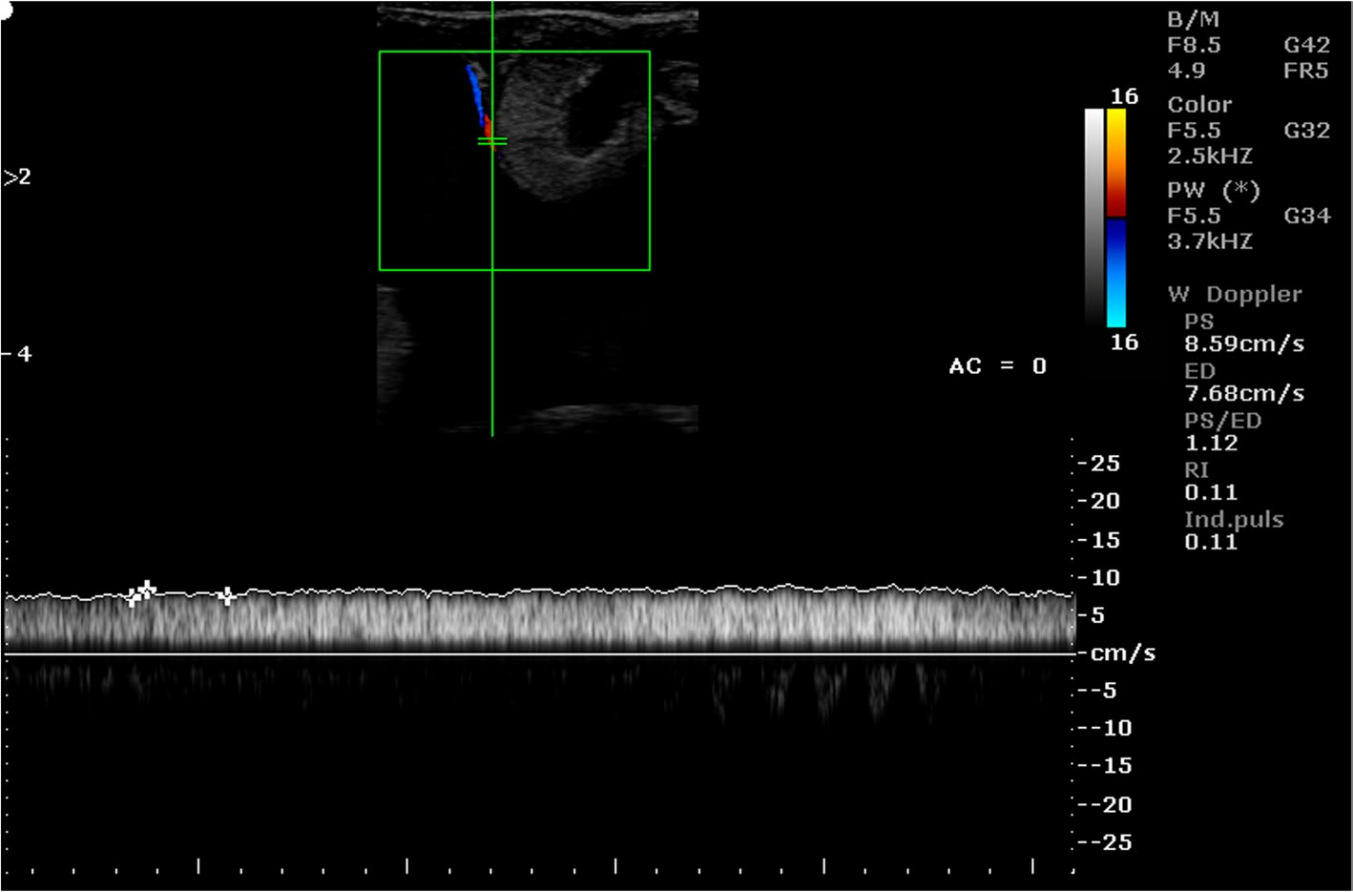
Color Doppler ultrasound image showing hemodynamic measurements from the caruncular vein of a sheep on day 70 of pregnancy (PS - peak systolic velocity; ED - end-diastolic velocity; RI - resistance index; Ind. puls - pulsatility index)

Also the PI values differed significantly depending on the location of the arterial vessel (Table 6). Table 7 shows the mean values of the Doppler parameters in the umbilical artery during consecutive stages of pregnancy. Whereas Tables 8 and 9 show the mean values of these parameters in the placental arteries. In the early stages of pregnancy, the values of PSV and EDV, regardless of the location of the vessel, were significantly lower than that in the later stages of pregnancy. On the other hand, PSV / EDV, RI and PI in the initial stages of pregnancy were significantly higher than that in the consecutive stages of pregnancy. Most of the Doppler parameters in the examined arterial vessels were significantly correlated with the day of pregnancy. The values of the correlation coefficients are presented in Table 10. Moreover, the analysis of variance showed that the values of Doppler parameters depended on both the period of pregnancy and the location of the artery. Example images of blood flow in umbilical arteries in different stage of pregnancy are presented in Figs. 5 and 6, and in placental arteries in Figs. 7, 8 and 9. The waveform of the Doppler spectrum in these vessels was of a pulsating nature, which in the umbilical artery and in the cotyledons, at the beginning of pregnancy, had a saw-tooth like appearance with only a systolic component but in the later stages of pregnancy, the diastolic breakdowns of the wave were also visible. Table 11 shows the mean values of the Doppler parameters of blood flow in the placental and umbilical veins in the period between 70 and 90 days of pregnancy. In the umbilical vein, all parameters were significantly higher than those in veins located in the cotyledons and caruncles. The Doppler spectrum in the veins was observed to be flat and wavy (Figs. 10, 11 and 12). In some of the imaged data, two spectra were visible simultaneously: one characteristic for the artery and the other for the vein.

**Table 7:** Mean (± SEM) values of Doppler parameters in the umbilical artery during consecutive stages of pregnancy in sheep (*n* = 16)

Period of pregnancy	Parameters				
	PSV (cm/s)	EDV (cm/s)	PSV/EDV	RI	PI
35–50 days	22.69 ± 1.34^Aa^	0.00^A^	NO	1.00^A^	3.01 ± 0.12^Aa^
55–70 days	22.26 ± 0.74^Aa^	1.93 ± 023^Ba^	8.67 ± 0.85^a^	0.95 ± 0.01^A^	2.79 ± 0.12^Aa^
75–90 days	26.15 ± 0.53^b^	4.25 ± 0.72^Bb^	9.56 ± 1.57^a^	0.78 ± 0.08^B^	1.65 ± 0.09^b^
95–110 days	27.79 ± 1.23^b^	6.91 ± 0.66^BC^	4.60 ± 0.42^b^	0.76 ± 0.03^B^	1.48 ± 0.13^B^
130–145 days	31.24 ± 1.43^B^	9.07 ± 0.92^BC^	4.22 ± 0.58^b^	0.70 ± 0.04^B^	1.17 ± 0.11^B^

A, a - indicate that mean values in the rows marked with different alphabets differ at *p* < 0.01 and *p* < 0.05, respectively

NO – incalculable value

**Table 8:** Mean (± SEM) values of Doppler parameters in the cotyledonary artery during consecutive stages of pregnancy in sheep (*n* = 16)

Period of pregnancy	Parameters				
	PSV (cm/s)	EDV (cm/s)	PSV/EDV	RI	PI
35–50 days	10.33 ± 0.17^Aa^	0.00^A^	NO	1.00^A^	2.26 ± 0.02^A^
55–70 days	13.18 ± 0.61^A^	0.04 ± 0.02^A^	12.03 ± 2.81^a^	1.00^A^	2.55 ± 1.14^A^
75–90 days	14.49 ± 0.76^Ab^	3.30 ± 0.34^B^	12.62 ± 4.42^a^	0.75 ± 0.03^B^	1.37 ± 0.10^Ba^
95–110 days	22.25 ± 0.79^B^	6.10 ± 0.41^C^	3.78 ± 0.33^b^	0.67 ± 0.03^B^	1.40 ± 0.06^Ba^
130–145 days	22.87 ± 3.32^B^	12.10 ± 1.13^D^	1.77 ± 0.48^b^	0.35 ± 0.12^C^	0.64 ± 0.17^Bb^

A, a - indicate that mean values in the rows marked with different alphabets differ at *p* < 0.01 and *p* < 0.05, respectively

NO – incalculable value

**Table 9:** Mean (± SEM) values of Doppler parameters in the caruncular artery during consecutive stages of pregnancy in sheep (*n* = 16)

Period of pregnancy	Parameters				
	PSV (cm/s)	EDV (cm/s)	PSV/EDV	RI	PI
35–50 days	11.07 ± 1.34^Aa^	2.10 ± 0.71^A^	0.95 ± 0.32^a^	0.87 ± 0.04^Aa^	1.66 ± 0.17^Aa^
55–70 days	14.34 ± 1.19^Aa^	7.91 ± 0.92^B^	3.99 ± 1.10^b^	0.36 ± 0.08^B^	0.73 ± 0.15^B^
75–90 days	17.86 ± 0.51^Ab^	9.13 ± 1.74^D^	4.84 ± 0.76^b^	0.42 ± 0.09^B^	0.79 ± 0.11^b^
95–110 days	26.20 ± 2.32^B^	10.32 ± 1.85^C^	3.23 ± 0.23^b^	0.56 ± 0.12^B^	0.91 ± 0.11^b^
130–145 days	29.18 ± 3.25^B^	12.21 ± 1.21^C^	2.42 ± 0.51^c^	0.52 ± 0.13^b^	0.89 ± 0.09^b^

A, a - indicate that mean values in the rows marked with different alphabets differ at *p* < 0.01 and *p* < 0.05, respectively

**Table 10:** Pearson correlation coefficients (r) between the day of pregnancy and the Doppler parameters in the arterial vessels of the placentomes and umbilical cord in sheep (*n* = 16)

Location of the vessel	Parameters	Correlation coefficients	Significance level
Umbilical cord	PSV	0.64	*P* < 0.01
	EDV	0.74	*P* < 0.01
	PSV/EDV	−0.04	NS
	RI	−0.72	*P* < 0.01
	PI	−0.82	*P* < 0.01
Cotyledon	PSV	0.66	*P* < 0.01
	EDV	0.90	*P* < 0.01
	PSV/EDV	−0.04	NS
	RI	−0.90	*P* < 0.01
	PI	−0.68	*P* < 0.01
Caruncle	PSV	0.56	*P* < 0.01
	EDV	0.60	*P* < 0.01
	PSV/EDV	0.21	NS
	RI	−0.57	*P* < 0.01
	PI	−0.48	*P* < 0.01

*NS* correlation is statistically insignificant

**Table 11:** Mean (± SEM) values of Doppler parameters in the umbilical cord and placentomal veins in the period between 70 and 90 days of pregnancy in sheep (*n* = 16)

Parameters	Location of vessel		
	umbilical cord	cotyledon	caruncle
PSV (cm/s)	18.51 ± 1.18^A^	12.97 ± 1.74^B^	10.77 ± 0.94^B^
EDV (cm/s)	13.44 ± 1.06^a^	11.50 ± 1,91^a^	8.95 ± 0.84^b^
PSV/EDV	1.44 ± 0.11^a^	1.16 ± 0.06^b^	1.22 ± 0.03
RI	0.32 ± 0.05^A^	0.12 ± 0.01^B^	0.18 ± 0.02^B^
PI	0.38 ± 0.06^Aa^	0.14 ± 0.02^B^	0.21 ± 0.03^b^

A, a - indicates that mean values in columns marked with different alphabets differ at *p* < 0.01 and *p* < 0.05, respectively

## Discussion

In this study, an early detection of pregnancy was performed using select ultrasound parameters and the Doppler indices of blood flow in the placental and umbilical vessels were determined. Pregnancy was detected at a time similar to that reported by other authors who used transrectal ultrasound for the early detection of pregnancy [1, 4, 24]. In these studies, the most frequently assessed parameters was the size of the uterus from a cross-sectional view. Images of enlarged, fluid-filled sections of the uterus are considered to be one of the earlier signs of pregnancy [25, 26]. On the other hand, a direct indicator of pregnancy is the presence of an embryo and an embryonic sac. In this study, embryos and embryonic sacs post mating were visible at times similar to those reported by other authors [26]. Additionally, it was found that the presence of corpora lutea on the ovaries may also be a helpful parameter.

Typically, during the sheep breeding season, the oestrus cycle lasts 17–19 days, with a short, 3–4 day follicular phase followed by a longer luteal phase [27]. Luteolysis begins around day 12, initiated by the uterine secretion prostaglandin F2α (PGF2α) [28]. On the other hand, the gestational corpus luteum is resistant to the luteolytic effects of this prostaglandin [5]. Therefore, the presence of the corpus luteum 17 days after mating may indicate that luteolysis has not occurred. Corpus luteum regression causes the extinction of activity and a reduction in the size of the corpus luteum [29]. On the other hand, in the study, the diameter of the corpus luteum between 17 and 24 days after mating was similar in size to that recorded by Rickard et al. [26] in sheep between the ovulation and the pre-implantation period. Hence, the identification of the corpus luteum/corpora lutea using B-Mode ultrasonography can be a very early and simple method to confirm successful mating in sheep. It seems that an important observation is also the appearance of the corpus luteum, which were identified as gray, echogenic, oval structures without a round anechoic central cavity. Indirectly, this may indicate that these are later stages of the development of the corpus luteum. Earlier stages of the corpus luteum are characterized by the presence of a central cavity in sheep and goats [26, 30]. In terms of the use of corpora lutea assessment for the early detection of pregnancy in sheep, interesting results were presented by Braganca et al. [25], Arashiro et al. [31] and Dall et al. [32]. The authors indicate the possibility of using Doppler ultrasound to assess the vascularization of the corpora lutea in the detection of pregnancy. The corpus luteum in pregnant sheep is the main source of progesterone from days 13 to 55 of pregnancy. After this time, this function is taken over by the placenta. Placental progesterone production is sufficient to maintain pregnancy in ovariectomized sheep from day 55 of gestation onwards [16]. This may explain the differences in the corpus luteum diameter noted in this study, which significantly decreased at the end of the first trimester of pregnancy. Another indicator of pregnancy is the presence of the placentomes [3, 33]. In this study, the first placentomes were observed between the first and second months of pregnancy. They were visible on the endometrial surface as areas of increased echogenicity in contrast to the hypoechoic uterine lumen. Kaşikçi et al. [33] observed the first placentomes on day 25, and Rickard et al. [26] on day 29 after insemination.

In this study, the umbilical cord was visualized for the first time around the 33rd day of pregnancy, similar to that reported by other authors [4]. Rickard et al. [26] visualized the umbilical cord already on the 23rd day of pregnancy, while Kumar et al. [34] identified free movement of the umbilical cord on the 39th day of pregnancy. At this stage, the cross-section of the umbilical cord was still small, but large enough to obtain a good measurement of its diameter. Measurements of the umbilical cord diameter at the beginning of pregnancy were also made by other authors [3, 35]. From the 35th day of pregnancy, it was also possible to estimate the blood flow velocity and other Doppler indices in the umbilical vessels. It should be emphasized that stress-free conditions were maintained in the study and no pharmacological agents were used. During the examination, the sheep were in standing position, which although poses difficulty for the examiner, did not cause anxiety in the animals, which is extremely important in this type of examination. The possibility of carrying out hemodynamic measurements of the umbilical artery in sheep without the use of anesthesia has been demonstrated in previous studies as well [3]. In the umbilical artery, peak systolic velocity increased as pregnancy progressed and this pattern was also found in other studies in sheep [3, 35–37]. In this study, as pregnancy progressed, the resistance and pulsatility indices decreased, which was also observed in other studies in sheep [3, 4, 36–38]. The end diastolic velocity was undetectable during the initial stages of pregnancy, while a gradual and progressive increase in this parameter was recorded from day 55 to the end of pregnancy. It is believed that the emergence of end diastolic velocity is related to the regularity of the fetal heart cycle and a decrease in the fetal heart rate [39].

As pointed out by Lemley [40], end diastolic velocities are clearly visible on day 90 and only poorly visible on day 60 of gestation in sheep. Elmetwally and Meinecke-Tillmann [41] did not observe umbilical artery end-diastolic velocity in goats and sheep until 12 weeks of gestation. Perhaps these differences are due to the location from where the measurements were done. It cannot be ruled out that the end diastolic velocity in the umbilical cord, at the beginning of pregnancy, is less pronounced the farther away from the fetus and closer to the placenta it is. This would explain the results from the cotyledonary arteries noted in this study, which together with the umbilical vessels form the placental-fetal system [16, 17]. In these vessels, EDV was undetectable or very poorly visible until mid-pregnancy. In this study, the blood flow in the umbilical vessels was examined near the abdominal insert, and as reported by Acharya et al. [42], the location of the umbilical cord being closer to the fetus may play an important role in regulating blood flow. It has also been suggested that the fetuses can regulate blood flow themselves by changing the diameter of the umbilical veins in the umbilical ring [42]. This may explain the differences between the flow parameters in the umbilical and cotyledonary vessels noted in this study. Moreover, in this study, the pulsatility and resistance indices in the caruncular vessels were significantly lower and therefore perhaps it can be assumed that this is required for the protection of the placenta. Saghian et al. [43] indicate that very high blood velocity and pressure can damage the delicate villi of the placenta, especially in the early stages of pregnancy. Placentomes are an integral part of the exchange between the maternal-placental and placental-fetal circulation and are supplied with blood from both the uterine and fetal sides [16, 17]. In addition, very high vascular resistance in the placenta may reduce gas exchange and nutrient delivery, which causes low birth weight and perinatal mortality [44, 45]. In the presented study, both in umbilical and placental arteries, the velocity of blood flow increased, and the indices of resistance and pulsatility decreased with the advancement of pregnancy.

The relationship between the gestational age and the hemodynamics of the umbilical cord and placental vessels noted in this study, is also confirmed by the significant correlation between the Doppler parameters and the day of pregnancy. Such changes in vascular hemodynamics are most likely offset by the significant increase in volumetric blood flow in umbilical and placental vessels with the progression of pregnancy [8, 46]. However, the study noted significant differences in the size of the examined Doppler indices between the caruncular arteries and the umbilical and cotyledonary arteries. The end diastolic velocity in the arteries of the caruncle was shown to be higher and the pulsatility and resistance indices lower than that in the umbilical and cotyledonary arteries. As reported by Riesen et al. [47], an increase in end diastolic velocity causes an increase in blood flow and a decrease in the resistance index. The differences noted in the study indicate that the hemodynamics in the placental vessels differs in the fetal and maternal parts of the placenta. Undoubtedly, it is related to the basic function of the placenta, which is the exchange of material between the maternal and fetal circulation [16, 17, 46]. Moreover, the placenta is also a metabolically active organ [9] and it therefore seems likely that a reduction in blood flow would first negatively affect the placenta, as this organ is the first to experience a reduced supply of nutrients and important substrates [7]. Doppler ultrasonography can distinguish between arterial and venous blood flow in umbilical vessels [48]. The blood flow in umbilical arteries is always pulsating, and the diameter of these vessels is larger than the diameter of the veins and increases with the progress of pregnancy [3].

In contrast, in the umbilical veins, the flow is continuous and not pulsating. The occurrence of pulsations may be associated with abnormal fetal development and perinatal complications, as observed in humans [49]. In this study, the Doppler spectrum showing the blood flow in the placental and umbilical veins was flat and wavy. In some of the imaged data, especially in the umbilical vessels, the two spectra were visible simultaneously: one characteristic for the artery and the other for the vein. Similar images were also presented in the umbilical veins of goats [34]. An almost constant flow velocity was demonstrated in the cotyledonary and caruncular veins. In contrast, in the umbilical vein, the peak systolic velocity and the end diastolic velocity were higher than that in the placental veins. Perhaps these higher values in the umbilical veins are due to their proximity to the fetus. As reported by Pennati et al. [50], umbilical vein flow velocity profiles vary along the umbilical cord. Venous flow is more susceptible to disturbances related to fetal movement than arterial flow [51]. In this study, a high value of diastolic velocity, close to the peak systolic velocity, was recorded in the examined venous vessels. The lack of the end diastolic velocity is believed to reflect an increased resistance in placental-fetal circulation, which may have adverse effects [52].

## Conclusion

The obtained results indicate that both the location of the arterial vessel in the placental-umbilical circulation and the gestational age have a significant impact on hemodynamic parameters. The results also provide new insight into blood flow in caruncular and cotyledonary arteries, which will contribute to a more holistic understanding of hemodynamic changes in sheep’s placenta. The studies conducted on haemodynamic parameters in venous umbilical vessels and placental vessels are preliminary studies in sheep, but may inspire further research in this field. Moreover, this research confirms the usefulness of transrectal ultrasonography in the early detection of pregnancy in sheep. We also indicate that the identification and imaging of the corpus luteum using B-mode ultrasound can be a very early and simple method of confirming effective mating in sheep.

## Material and methods

### Animals and the layout of the experiment

The study was carried out on 16 Suffolk sheep kept on an organic sheep farm in the Experimental Station of the National Research Institute of Animal Production in Kołbacz (Poland: latitude 53′30″ N). The sheep were kept in the pastures and indoor systems. The feeding was carried out in accordance with the standards adopted for this species, which is based on pasture green, other roughage and concentrated fodder, depending on the season. The animals had constant access to water and salt licks. The examined sheep are healthy multiparous females, aged 3 to 4 years, with an even body weight (55–60 kg). The sheep were mated during their natural breeding season (September). The estrus was detected using a teaser ram, and mating was hand-service. The duration of pregnancy was determined on the basis of the date of mating. The effectiveness of mating was examined with the transrectal ultrasound (USG scanner EDAN U50, linear probe with 4 MHz frequency). After delivery, the date of conception was confirmed retrospectively by assuming that the pregnancy lasted 148 days [3]. The following parameters were taken into account in the early detection of pregnancy: the size and echogenicity of the uterine cross-section, the presence of corpora lutea on the ovaries, and the presence of the embryo and the gestational sac in the uterus. The time when the placentomes and umbilical cord were first visible was also noted. These parameters were assessed from the 17th day after mating at intervals of several days. In order to measure the blood flow in the umbilical arteries and the placentomes, the study was started on day 35 and continued throughout the pregnancy at intervals of several days. These analyses included 5 periods: 1st period (35–50 days), 2nd period (55–70 days), 3rd period (75–90 days), 4th period (95–110 days) and 5th period (130–145 days). The blood flow in the veins was measured from day 70 to day 90 of pregnancy. In order to eliminate the possible influence of the size of the litter on the examined parameters, only single pregnancies were analyzed, which were diagnosed during transrectal examination and confirmed during delivery.

### Ultrasound examination

Ultrasound examinations were performed on pregnant sheep that had not been previously sedated. The examination was always performed by the same experienced and trained operator, in a quiet and dimly lit room. The ultrasound examination was performed using an ultrasound scanner (USG scanner EDAN U50) equipped with a linear probe with a frequency of up to 4 MHz (Model, V742UB) and a sector probe with a frequency of up to 5 MHz (Model, C352UB).

**Fig. 13:**
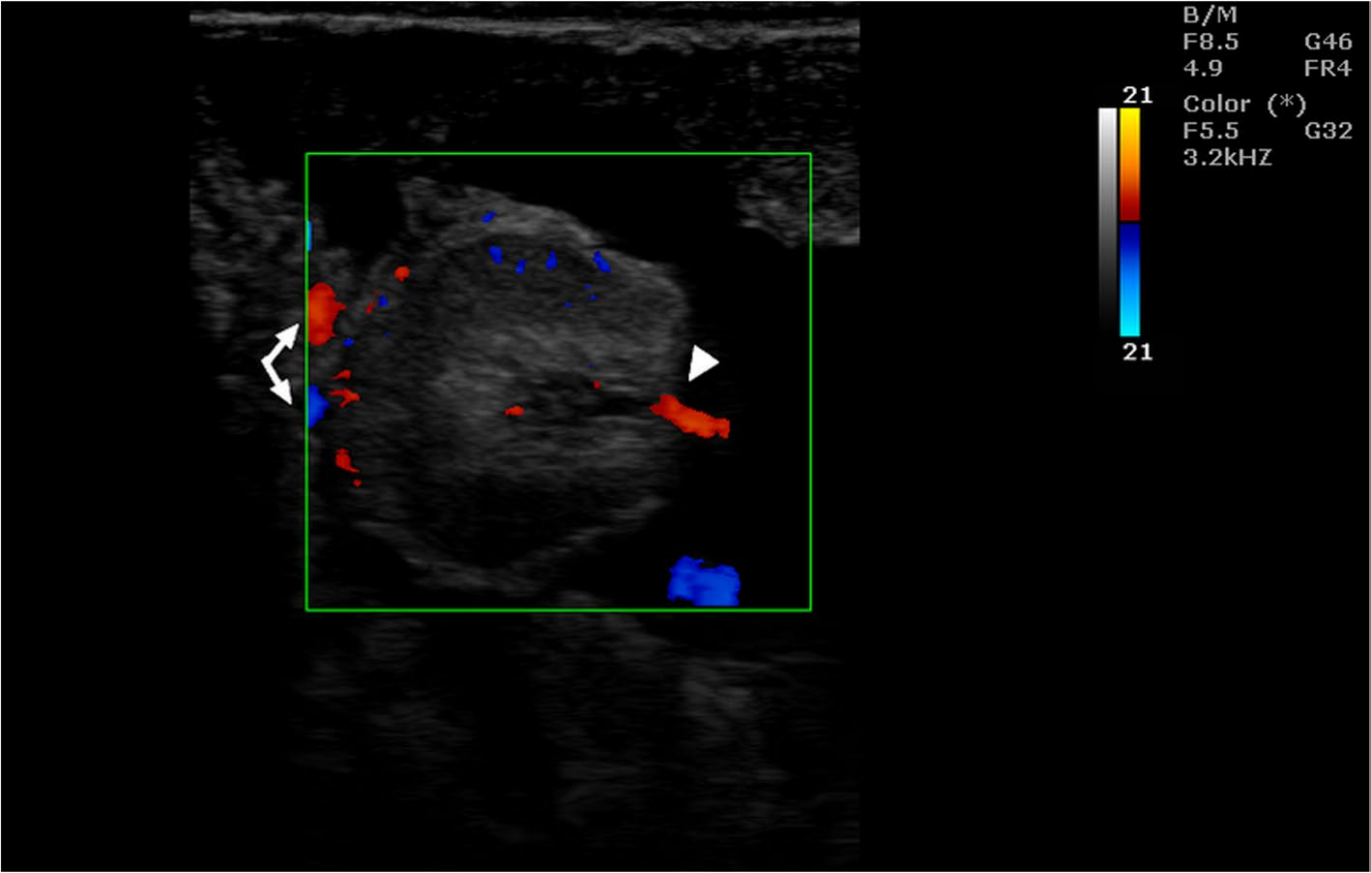
Color Doppler ultrasound of a sheep placenta on day 70 of pregnancy. Arrows - vessels of caruncle, arrowhead - vessels of cotyledon

In the initial stages of pregnancy, ultrasound was performed transrectally, and in subsequent stages of pregnancy, sheep were examined transabdominally. Sheep were in standing position during the ultrasound examination. Prior to insertion of the probe, fecal pellets were removed from the rectum. About 20 ml of ultrasound gel was introduced rectally for better visualization of the organs [3, 14, 32]. Prior to transabdominal examination, the inguinal and caudal abdomen were shaved and the skin was cleansed with soap and water. A sufficient amount of transmission gel was applied before the ultrasound examination. Each sheep was examined for the free part of the umbilical cord near the abdominal insertion and for 5 randomly selected placentomes, which were scanned using ultrasound in the B mode. Color Doppler was used to identify arterial and venous vessels in the umbilical cord, cotyledons and caruncles (Fig. 13). Each time, after locating the blood vessels, the blood flow in the arteries and veins was measured using the Doppler pulse wave ultrasound technique. The following Doppler parameters were determined: peak systolic velocity (PSV), end diastolic velocity (EDV), PSV/EDV ratio, resistance index [RI = (PSV-EDV)/PSV] and pulsatility index [PI = (PSV-EDV)/mean velocity]. The flow angle during the examination was kept as close to 0 degrees as possible, with appropriate angle adjustments being made when necessary. Doppler imaging for each sheep lasted from 20 to 30 minutes. Doppler measurements of blood flow in the arteries were made on 5–7 continuous, regular waves of the Doppler spectrum, while venous flows took into account the entire visible spectrum. Measurements were not recorded during maternal and fetal movements. In the event of any signs of distress or tachypnea, the examination was discontinued and repeated at a later time.

### Statistical analysis

The obtained results were further submitted for statistical analysis. The results concerning the ultrasound biometric parameters assessed in the early period of pregnancy are represented as mean ± SD, while the results of the Doppler parameters are represented as mean ± SEM. Differences between the means of individual groups were analyzed using the post hoc test. Duncan’s multiple range test was used to verify the significance of differences at *P* < 0.01 and *P* < 0.05. In order to determine the influence of the gestation period and the location of the blood vessel on the blood flow parameters, the multivariate analysis of variance (ANOVA) was performed, where the grouping variable was the gestational age and the location of the arterial vessel, and the dependent variable was the Doppler parameter. The F-test was used to determine the significance level. The correlations between the examined parameters and the day of gestation were calculated using the Pearson rank correlation coefficient (r). Statistical analyses were conducted using the STATISTICA version 13.3, Stat Soft, Poland.

## Data Availability

The datasets used and analysed during the current study are available from the corresponding author on reasonable request.
